# Kinetics of cytomegalovirus and Epstein-Barr virus DNA in whole blood and plasma of kidney transplant recipients: Implications on management strategies

**DOI:** 10.1371/journal.pone.0238062

**Published:** 2020-08-25

**Authors:** Tiziana Lazzarotto, Angela Chiereghin, Antonio Piralla, Dino Gibertoni, Giulia Piccirilli, Gabriele Turello, Giulia Campanini, Liliana Gabrielli, Cristina Costa, Giorgia Comai, Gaetano La Manna, Luigi Biancone, Teresa Rampino, Marilena Gregorini, Francesca Sidoti, Gabriele Bianco, Maria Vittoria Mauro, Francesca Greco, Rossana Cavallo, Fausto Baldanti, on behalf of the AMCLI-GLaIT

**Affiliations:** 1 Microbiology Unit, Laboratory of Virology, Department of Specialized, Experimental, and Diagnostic Medicine, St. Orsola Polyclinic, University of Bologna, Bologna, Italy; 2 Molecular Virology Unit, Microbiology and Virology Department, Foundation IRCCS Polyclinic San Matteo, Pavia, Italy; 3 Hygiene and Biostatistics Unit, Department of Biomedical and Neuromotor Sciences, University of Bologna, Bologna, Italy; 4 Microbiology and Virology Unit, A.O.U. “Città della Salute e della Scienza di Torino”, University of Turin, Turin, Italy; 5 Nephrology, Dialysis and Renal Transplant Unit, Department of Specialized, Experimental, and Diagnostic Medicine, St. Orsola Polyclinic, University of Bologna, Bologna, Italy; 6 Nephrology, Dialysis and Kidney Transplantation Unit, Department of Medical Sciences, A.O.U. "Città della Salute e della Scienza di Torino", University of Turin, Italy; 7 Nephrology, Dialysis and Transplant Unit, Foundation IRCCS Polyclinic San Matteo, Pavia, Italy; 8 Department of Microbiology and Virology, SS Annunziata Hospital, Cosenza, Italy; University of St Andrews, UNITED KINGDOM

## Abstract

This retrospective multicenter cohort study investigated the kinetics (ascending and descending phases) of cytomegalovirus (CMV) and Epstein-Barr virus (EBV)-DNA in whole blood (WB) and plasma samples collected from adult kidney transplant (KT) recipients. CMV-DNA kinetics according to antiviral therapy were investigated. Three hundred twenty-eight paired samples from 42 episodes of CMV infection and 157 paired samples from 26 episodes of EBV infection were analyzed by a single commercial molecular method approved by regulatory agencies for both matrices. CMV-DNAemia followed different kinetics in WB and plasma. In the descending phase of infection, a slower decay of viral load and a higher percentage of CMV-DNA positive samples were observed in plasma versus WB. In the 72.4% of patients receiving antiviral therapy, monitoring with plasma CMV-DNAemia versus WB CMV-DNAemia could delay treatment interruption by 7–14 days. Discontinuation of therapy based on WB monitoring did not result in relapsed infection in any patients. Highly different EBV-DNA kinetics in WB and plasma were observed due to lower positivity in plasma; EBV positive samples with a quantitative result in both blood compartments were observed in only 11.5% of cases. Our results emphasize the potential role of WB as specimen type for post-KT surveillance of both infections for disease prevention and management.

## Introduction

Cytomegalovirus (CMV) and Epstein-Barr virus (EBV) have a central role in kidney transplant recipients (KTRs) [1]. Specifically, CMV is associated with detrimental direct and indirect effects (such as chronic allograft nephropathy and/or allograft loss) [2]. EBV has a pivotal role in the pathogenesis of post-transplant lymphoproliferative disorder (PTLD), a life-threatening complication [3]. Early detection of viral replication and optimal treatment of active infection may significantly impact transplant outcomes [2,4]. However, which of the blood compartments–whole blood (WB) or plasma–is optimal for testing is unclear [5]. In solid organ transplant (SOT), kinetics analyses have never been performed of CMV and EBV-DNAemia in WB and plasma using a single, approved molecular method for quantifying CMV and EBV-DNA in both blood matrices. In a previous study [6], we investigated the kinetics of both CMV and EBV-DNAemia in WB and plasma collected from pediatric and adult allogeneic hematopoietic stem cell transplant (HSCT) recipients using a CE-marked and FDA-approved automated molecular method. These data demonstrated that CMV and EBV-DNAemia follow different kinetics in the two blood compartments in HSCT [6]. In the present study, we investigated the kinetics (ascending and descending phases) of both viruses in WB and plasma samples collected from adult KTRs. These observations may impact monitoring, prophylactic and therapeutic management strategies for these infections.

## Materials and methods

### Study design

In this retrospective non-interventional multicenter cohort study, adult patients who underwent KT at four Italian transplant centers (Bologna, Pavia, Turin and Cosenza) in the period between June 2014 and October 2015 were enrolled. At each transplant center, routine virological surveillance for CMV and EBV was performed on EDTA-anticoagulated WB samples using local molecular assay following the same time-schedule. In particular, virological measurements were performed weekly for 3 months after transplant, twice a month from months 3–6, and then monthly from months 6–12. Afterwards, blood samples were analyzed if clinically indicated. Clinical intervention strategies for CMV and EBV infections (Table 1) were guided by the results obtained. Mode of storage and selection criteria of the samples to investigate, using a single automated quantitative commercial PCR assay (see below), were those previously reported for allogeneic HSCT recipients [6]. Of note, in the four centers, samples were consistently handled in terms of collection, transport and storage conditions (i.e. timing and temperature of storage as well as time elapsed between blood collection and separation of plasma) in order to minimize the quantification variability due to the pre-analytical phase.

**Table 1:** CMV and EBV prevention strategies according to transplant center.

	BOLOGNA	PAVIA	TURIN	COSENZA
**CMV Prophylaxis** anti-CMV agent^**a**^– dose–duration	R+, D+/R- VGCV– 900 mg once daily– 3 (R+) and 6 (D+/R-) months post-TX	NO	D+/R- and BSX induction: VGCV– 900 mg once daily– 6 months post-TX R+, R- and ATG induction: VGCV– 900 mg once daily– 1–2 (R+) and 6 (R-) months post-TX	D+/R- VGCV– 900 mg once daily– 3–6 months post-TX
**CMV Preemptive** Cut-off viral DNA levels anti-CMV agent^**a**^ –dose and duration	R+, D+/R- > 100,000 copies/mL WB VGCV^**b**^– 900 mg twice daily	R+, D+/R- > 300,000 copies/mL WB VGCV– 900 mg twice daily or GCV^**b**^ i.v.– 5 mg/kg once daily	R+ and BSX induction > 5,000 copies/mL WB VGCV^**b**^– 900 mg twice daily –	R+ > 5,000 copies/mL WB GCV^**b**^ i.v.– 5 mg/Kg once daily
**EBV** Cut-off viral DNA levels drug–dose–duration	R+, R- > 50,000 copies/mL WB or when a rapid increase of blood viral load was detected Reduction of the IS Anti-CD20 monoclonal antibody rituximab^**c**^– 4 administrations at dose of 375 mg/m^2^/week	R+, R- > 5,000 copies/mL WB abdominal ultrasound, chest x-ray, head and neck x-ray > 100,000 copies/mL WB Reduction of the IS Anti-CD20 monoclonal antibody rituximab^**c**^ – 4 administrations at dose of 375 mg/m^2^/week	No patients enrolled	No patients enrolled

D: donor; R: recipient; +: positive; -: negative; VGCV: valganciclovir; GCV: ganciclovir; BSX: basiliximab; ATG: anti-thymocyte immunoglobulin; TX: transplant; WB: whole blood; i.v.: intravenous; IS: immunosoppressive therapy.

^**a**^The dosages of the antiviral drugs during therapy were renal function adjusted.

^**b**^Until at least two whole blood samples resulted CMV-DNA negative.

^**c**^When reducing immunosuppression alone is not sufficient to control EBV-DNAemia or when symptoms suggested EBV-related disease.

### Molecular assays

Extraction and quantification of CMV and EBV-DNA in paired WB and plasma samples were performed, as previously described [6], using the commercial automated QIAsymphony RGQ System (QIAGEN, Hamburg, Germany) and the *artus*^®^ QS-RGQ kits on the Rotor-Gene Q instrument (QIAGEN), respectively. Using this certified method, results can be reported as International Unit (IU)/mL, as recommended by the 2018 international consensus guidelines on the management of CMV in SOT [7]. However, recent studies have shown that some factors such as the amplicon size and the fragmentation of circulating viral DNA may impact the commutability of CMV and EBV quantitative standards [8,9].

In our study, aiming to compare the kinetics of CMV and EBV-DNA in two different blood compartments, we retained to report the results as copies/mL.

### Statistical analysis

In order to include in the analyses the positive samples with values below the lower limit of quantification (LLoQ) of the assay, the data were transformed to approximately half the LLoQ value: WB <1,000 copies/mL were transformed to 500 copies/mL in both CMV and EBV assays; plasma <79.4 copies/mL were transformed to 40 copies/mL in CMV and plasma <316 copies/mL to 150 copies/mL for the EBV assay. The imputation of half the LLoQ to the LLoQ values is a procedure already proposed to manage not quantifiable samples in other settings (e.g. pharmacokinetic analyses) [10]. All analyses were performed using log_10_ copies/mL and for this reason negative samples were assigned an arbitrary value of 1 copy/mL. For each infection episode and each blood compartment, the week in which the peak viral load was detected was marked as week 0, and values of viral load in the five preceding and following weeks were retained for the analysis of kinetics. Viral load distributions in the two blood compartments were compared by means of Spearman’s correlation. WB peak values and the corresponding plasma values were compared using Wilcoxon matched-pair test. Viral load kinetics were first described by estimating the linear slopes of the ascending and descending phases, and testing each pair of slopes against the null hypothesis of equality using the Wald test. Subsequently, a repeated-measures ANOVA with infection episodes nested within compartments was performed on ascending and descending phases separately. This method allowed to verify whether viral load changed over time, taking into account the non-independence of episodes between compartments. Model-estimated viral loads from week -5 to week +5 for each compartment were obtained, as well as contrasts of viral load at adjacent weeks within the same compartment and of the two compartments at every week.

All these analyses were conducted on the two overall samples of CMV and EBV infections and subsequently, only for CMV infection, on patient subgroups based on antiviral therapy treatment, immunosuppressive regimens and underlying disease. The immunosuppressive regimens used in the study cohort are reported in S1 Table. Tests were considered significant for p-values ≤ 0.05. All analyses were performed using Stata v. 15.1.

### Ethics

The study was approved by all four transplant centers’ Ethics Committee (i.e. Comitato Etico Indipendente di Area Vasta Emilia Centro, Comitato di Bioetica di Pavia, Comitato Etico Interaziendale A.O.U. Città della Salute e della Scienza di Torino—A.O. Ordine Mauriziano—A.S.L. Città di Torino Comitato Etico and Comitato Etico Regione Calabria Sezione Area Nord). All patients provided written informed consent.

## Results

### Study cohort

Sixty-four adult single KTRs met the study selection criteria, for a total of 68 infection episodes (42 of CMV infection and 26 of EBV infection), as both first and second episode of CMV and EBV infection were identified for three and one patients, respectively. Clinical and virological characteristics of the study population are reported in Table 2. In particular, six episodes of primary CMV infection (6/42 [D+/R-]; 14.3%) and 1 episode of primary EBV infection (1/26 [D?/R-]; 3.8%) were included in the study cohort. CMV-related symptoms were observed in 18 (18/42; 42.9%) active CMV infection episodes. Viral syndrome with: i) leukopenia (i.e. leukocyte count < 4,000 cells/μl, n = 12 [9 D+/R+, 2 D-/R+, 1 D+/R-]; 66.7%), ii) gastrointestinal symptoms (n = 3 [D+/R+]; 16.7%), iii) fever and malaise (n = 1 [D+/R+]; 5.5%), and tissue-invasive disease (n = 2 [D+/R+]; 11.1%) were observed. No EBV-related symptoms were observed. The total number of paired samples investigated for the search of CMV and EBV-DNA was 328 and 157, respectively; the median number of sequential paired samples tested per infection episode was 7 (range, 7–11). Due to the low number of second infection episodes included in the study, viral load kinetics in WB and plasma were analyzed regardless of the type (first episode versus relapse) of infection. Similarly, the effect of everolimus on viral kinetics could not be investigated. The study cohort also comprised a small control group to confirm the 100% assay specificity reported by the manufacturer. Specifically, 15 paired samples from 3 patients negative for CMV infection and 10 paired samples from 2 patients negative for EBV infection were retrospectively investigated.

**Table 2:** Clinical and virological characteristics of the study cohort with active CMV or EBV infection.

	CMV infection	EBV infection
**No. of patients** (Male/Female)	39 (19/20)	25 (20/5)
**No. of active infection episodes**		
First infection episode	37	18
Relapse of infection	5	8
Total	42	26
**No. of samples**		
Whole blood	328	157
Plasma	328	157
Total	656	314
**Antiviral/Anti-CD20 monoclonal antibody therapy—No. of active infection episodes**		
Treated	34^**a**^	0
Not treated	8^**b**^	26
**Clinical course of active infection episodes**		
Asymptomatic	24	26
Symptomatic	18	0
**D/R Serostatus–No. of patients**		
D+/R+	25	6
D+/R-	6	0
D-/R+	4	0
D?/R+	4	18
D?/R-	0	1
D-/R-	0	0
**Underlying disease–No. of patients**		
Malformative nephropathy	15	4
End stage renal disease	12	9
Proteinuric nephropathy	8	3
Vascular nephropathy	4	9
**Immunosuppressive therapy, Induction–Maintenance—No. of active infection episodes**		
BSX–FK + steroids + MMF	32	15
ATG–FK + steroids + MMF	6	7
ATG–FK + steroids + EVR	2	3
BSX–FK + steroids + EVR	2	1

D: donor; R: recipient; D?: donor not available; +: positive; -: negative; BSX: basiliximab; FK: tacrolimus; MMF: mycophenolate mofetil; ATG: anti-thymocyte immunoglobulin; EVR: everolimus.

^**a**^First infection episode (n = 29)/Relapse of infection (n = 5).

^**b**^All first infection episodes.

### CMV infection

#### CMV-DNA kinetics in WB and plasma

**Fig 1:**
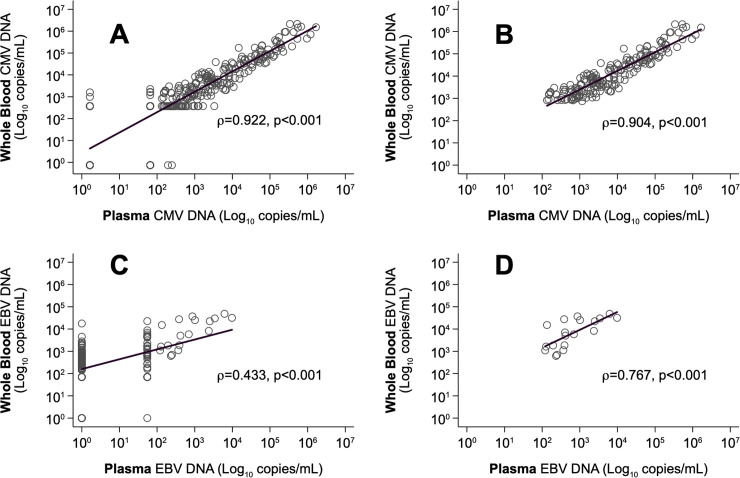
Spearman correlation between viral DNA levels evaluated in plasma and WB samples of KTRs. CMV-DNA values, A: all samples (n = 328 paired samples); B: positive samples with a quantitative result (n = 186 paired samples). EBV-DNA values, C: all samples (n = 157 paired samples); D: positive samples with a quantitative result (n = 18 paired samples).

**Fig 2:**
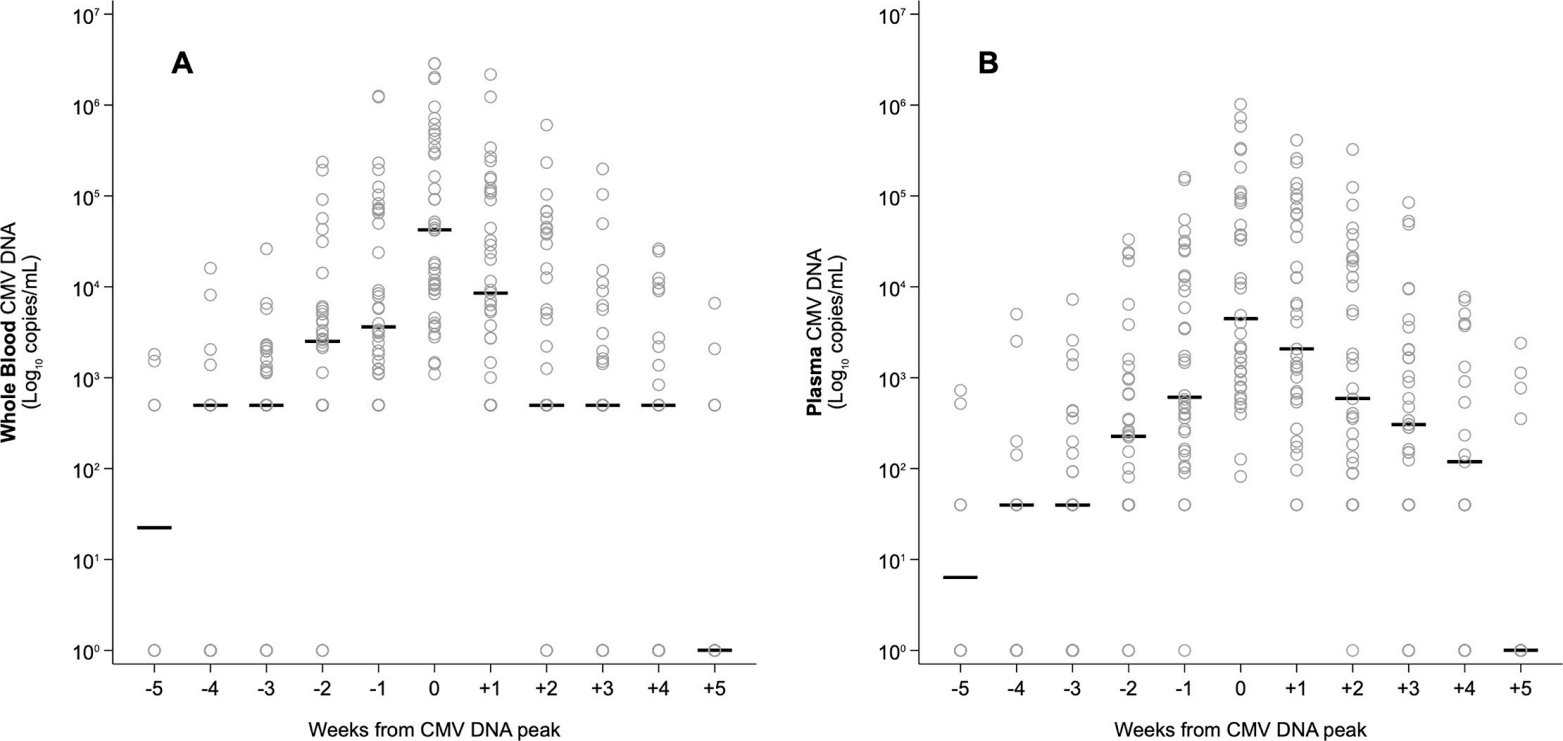
Distribution per week of CMV-DNA values in WB and plasma samples with respect to CMV-DNA peak in WB, placed at T0. A: WB samples. B: plasma samples.

Among the 328 samples analyzed, 26 (7.9%) samples showed discordant results between WB and plasma. In detail, 14/26 (53.8%) samples were positive in WB (11 samples were positive but below the LLoQ of the assay and 3 samples were positive with a quantitative result) and negative in plasma. The remaining 12 (46.2%) samples tested negative in WB and positive in plasma (10 were positive but below the LLoQ and 2 samples were positive with a quantitative result). CMV-DNA levels in WB and plasma were highly correlated both when including all samples (Spearman’s correlation ρ = 0.922; p<0.001, Fig 1A) and the samples with a positive quantitative result in both blood compartments (Spearman’s ρ = 0.904; p<0.001, Fig 1B). All paired samples from CMV-negative patients resulted DNA negative. The distribution of CMV-DNA values with respect to WB peak time (T0) was similar in the two blood compartments (Fig 2A and 2B). All plasma samples were CMV-DNA positive at T0 and in 59.5% of cases (25/42 active infection episodes) CMV-DNA peak levels were reached simultaneously in the two blood compartments; peak levels in plasma were reached one week before the peak in WB in 2/42 (4.8%) episodes, and following the peak in WB in the remaining 15/42 (35.7%) episodes (among them, 11 were at week +1). However, median WB CMV-DNA peak values (44,538 copies/mL, range, 1,100–2,862,000) were higher than their corresponding plasma values (4,452 copies/mL, range, 82–1,017,000) and differed significantly (Wilcoxon matched-pair test: z = 15.48, p<0.001). In addition, 12/14 samples (85.7%) tested positive in WB and negative in plasma were detected in the 5 weeks prior to the peak; these samples were from 10 first infection episodes. Concerning the descending phase, a significant difference between the results obtained in WB and plasma was observed. Specifically, a higher percentage of negative or low positive (below the LLoQ) samples was detected in WB than in plasma at week +1 (25.6% vs. 7.7%; p = 0.013), +2 (50% vs. 23.7%; p = 0.031) and +3 (61.1% vs. 30.5%; p = 0.017).

**Fig 3:**
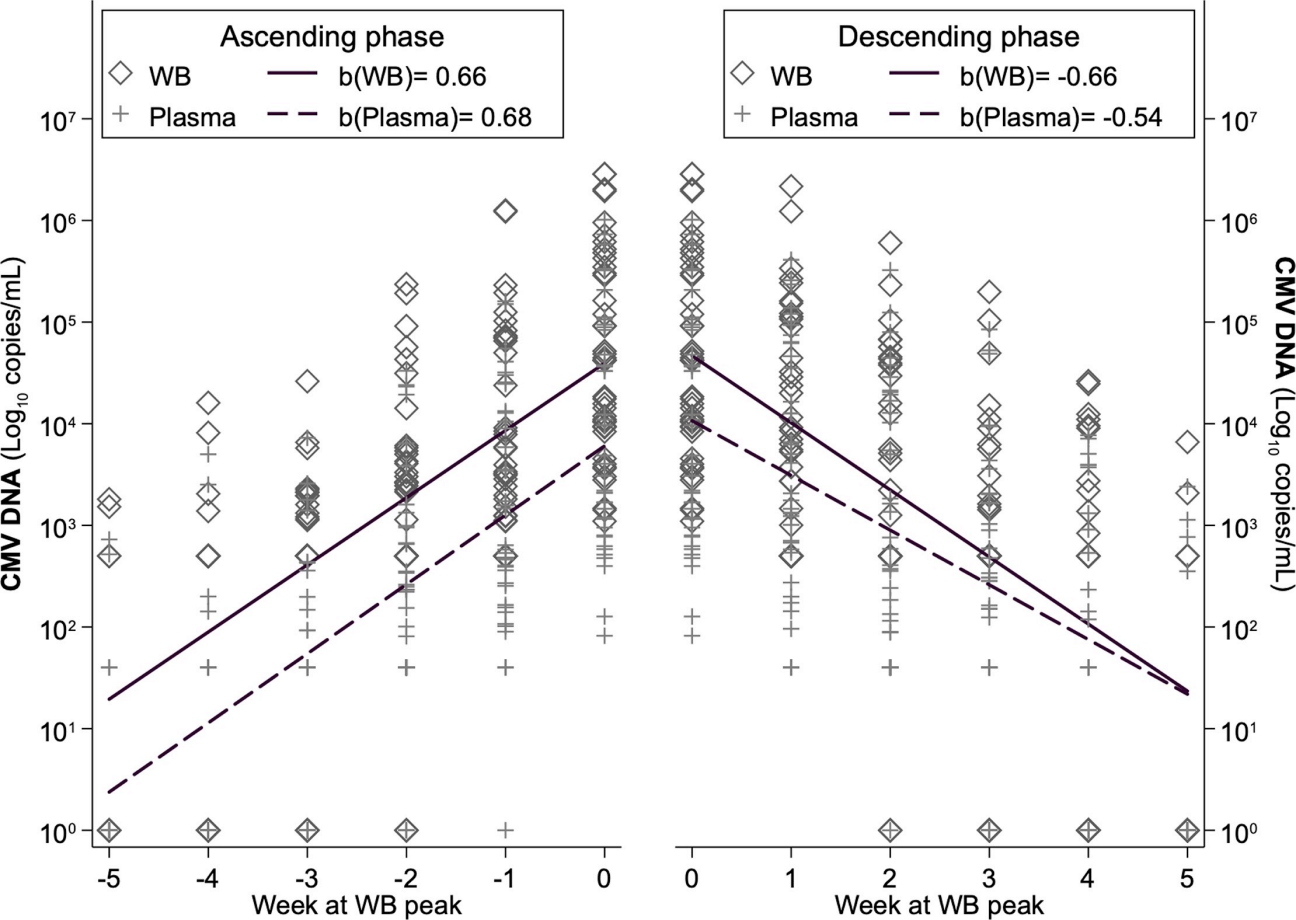
Ascending and descending linear slopes of viral load in WB and plasma, all samples.

Viral load in the ascending phase was constantly higher in WB than in plasma, producing similar linear slopes: β = 0.661 for WB and β = 0.681 for plasma (Wald test: χ^2^ = 0.27, p = 0.600) (Fig 3). On the contrary, in the descending phase, viral load values in WB progressively approached those of plasma, and as a result, the WB slope (β = -0.660) was steeper than the plasma slope (β = -0.540) with a significant difference (Wald test: χ^2^ = 16.01, p<0.001).

**Fig 4:**
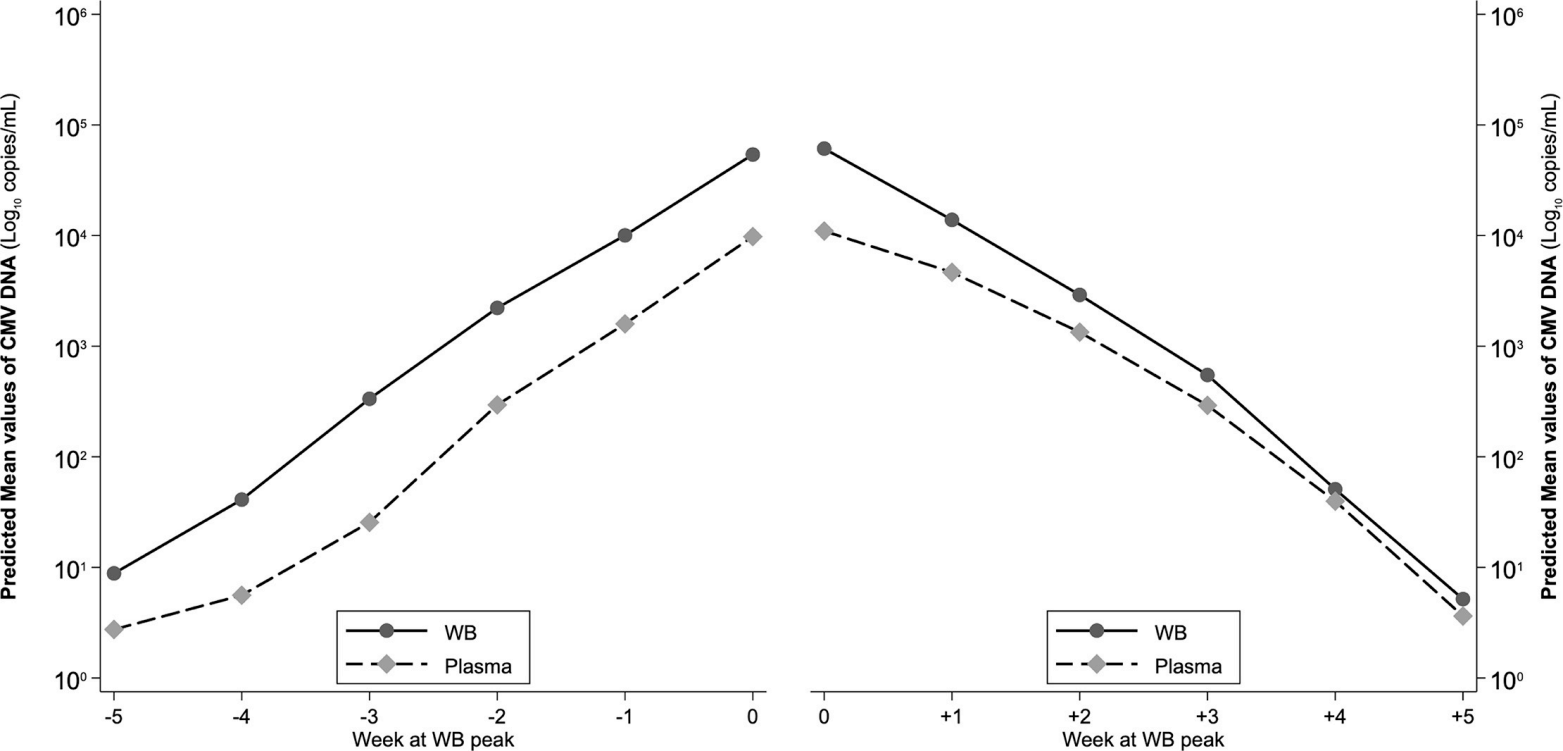
ANOVA-estimated ascending and descending trajectories of viral load in WB and plasma, all samples.

The repeated-measures ANOVA conducted on the ascending phase provided a significant difference between CMV-DNA WB and plasma values (F = 8.69, p = 0.004), a significant modification of viral load with time (F = 131.07, p<0.001), while the interaction between compartment and time was not significant (F = 0.58, p = 0.647 with Greenhouse-Geisser correction), indicating that the difference estimated between WB and plasma did not modify with time (Fig 4). The estimated increase of viral load between adjacent measurements in the same compartment was significant in both compartments for each pair of measurements, with the exception of the -5 week to -4-week increase. Correspondingly, contrasts between compartments at the same week were significant at all weeks except for week -5, meaning that from week -4 to week 0 viral load in WB was always higher than in plasma.

In the descending phase, due to the steeper slope of WB, viral load in WB was significantly higher than in plasma only at week +1 (p = 0.004) and at week +2 (p = 0.043), while from week +3 onwards, estimates of viral load in the two compartments did not differ significantly (Fig 4). This resulted in no overall difference between WB and plasma values in the descending phase (F = 1.43, p = 0.256), a significant difference of viral load with time (F = 160.82, p<0.001) and a non-significant interaction between compartment and time (F = 1.61, p = 0.198 with Greenhouse-Geisser correction), because from week +3 onwards the two slopes are almost parallel. The decrease of viral load within the same compartment was significant between each pair of weeks, for both compartments.

Finally, higher levels of CMV-DNA in WB than in plasma in the ascending phase of infection was also observed in the 12 patients with leukopenia; in particular, a median difference of 0.77 log_10_ copies/mL (range, 0.17–1.74) between the two blood compartments was observed (Wilcoxon matched-pair test: z = -4.93, p<0.001).

### CMV-DNA kinetics in WB and plasma according to antiviral therapy, immunosuppressive regimens and underlying disease

**Fig 5:**
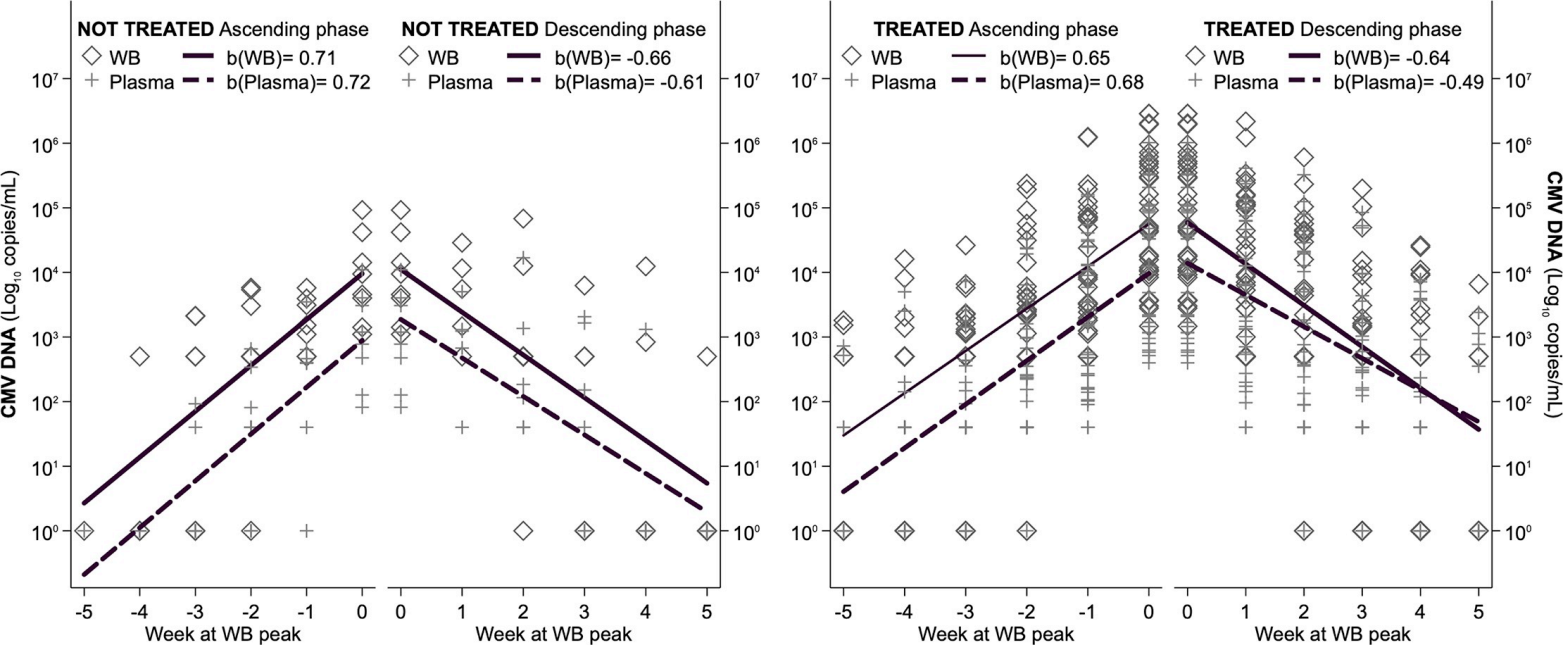
Ascending and descending linear slopes of viral load in WB and plasma, for treated with antiviral therapy and not-treated patients.

**Fig 6:**
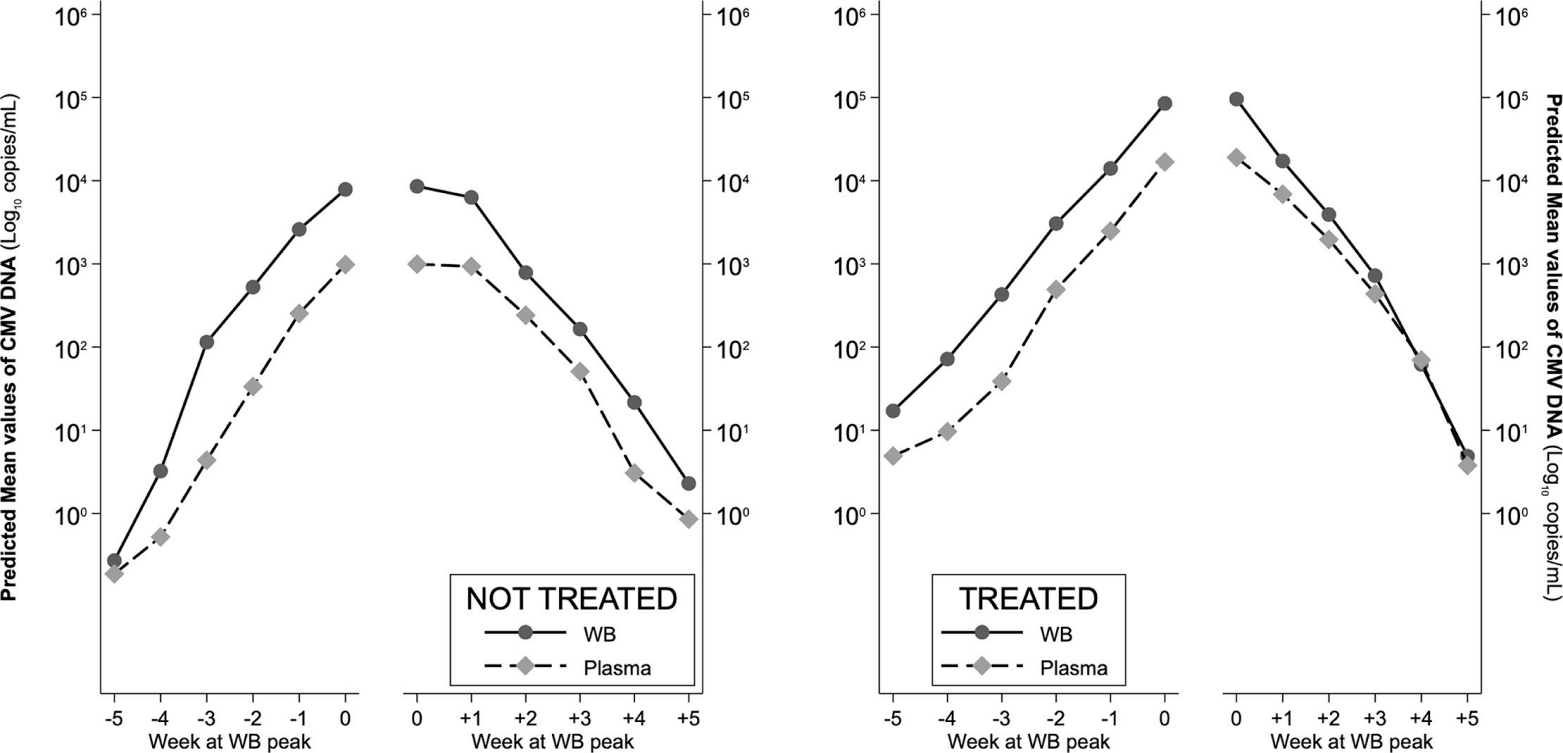
ANOVA-estimated ascending and descending trajectories of viral load in WB and plasma, for treated with antiviral therapy and not-treated patients.

According to monitoring in WB, 29 patients received antiviral therapy during the first infection episode (Table 2). The slopes of ascending viral load were similar in treated and not-treated patients, with viral load constantly higher in WB; in the descending phase, slopes were very similar in not-treated patients, i.e. viral load remained constantly higher in WB, while in treated patients, the slopes very quickly converged because of a steeper decline of viral load in WB (Wald test: χ^2^ = 23.90, p = <0.001) (Fig 5). This pattern was confirmed by the repeated-measures ANOVA (Fig 6) which overall assessed, for the ascending phase significant differences between compartments (higher viral load in WB, F = 7.54, p = 0.007), between treatment (higher viral load in treated, F = 46.56, p<0.001), along time (increasing viral load from week -5 to week 0, f = 77.30, p<0.001) and non-significant interactions of compartment and treatment with time, indicating that the differences found remained constant during all the ascending phase. Specifically, viral load differed significantly between compartments in treated from week -4 onwards and in non-treated from week -3 onwards. On the descending phase, significant differences were confirmed along time (F = 104.54, p<0.001) and between treatment (higher viral load in treated, F = 41.45, p<0.001), while viral load was not different between compartments (F = 1.93, p = 0.168). The estimated differences in viral load were significant between compartments only at time +1 for treated (F = 5.29, p = 0.022) and always failed to reach significance for non-treated (F = 2.97, p = 0.086 at time +1), probably also because of their limited sample size.

Notably, if CMV monitoring had been performed in plasma, in the 72.4% of cases (21/29 patients) the treatment interruption would have been delayed 7–14 days due to residual plasma CMV-DNAemia signals. None of these patients developed relapse of infection during the post-transplant follow-up period of at least one-year duration. In the remaining 27.6% of the cases (8/29 patients), CMV-DNA negativity was achieved simultaneously in the two blood compartments and three relapse episodes were observed within one month after the resolution of the first infection episode.

Consistent results were found analyzing the viral load kinetics in subgroups defined by immunosuppressive regimens (S1 and S2 Figs) or underlying disease (S3 Fig): in each subgroup, in the ascending phase the slopes of WB and plasma were almost parallel, while in the descending phase the WB slope was steeper. The slightly different pattern found in patients with vascular nephropathy is likely due to the limited size of this subgroup.

### EBV infection

#### EBV-DNA kinetics in WB and plasma

Among the 157 samples analyzed, 84 (53.5%) samples showed discordant results in the two blood compartments. In detail, 83 (98.8%) samples were positive in WB (66 samples were positive with a quantitative result and 17 were positive but below the LLoQ of the assay) and negative in plasma; one sample (1.2%) resulted negative in WB and positive but below the LLoQ of the assay in plasma. The correlation between WB and plasma values was 0.433 (p<0.001) when all samples were evaluated (Fig 1C) and 0.767 (p<0.001) when only the positive samples with a quantitative result in both the matrices (Fig 1D) were compared. All paired samples from EBV-negative patients resulted DNA negative.

**Fig 7:**
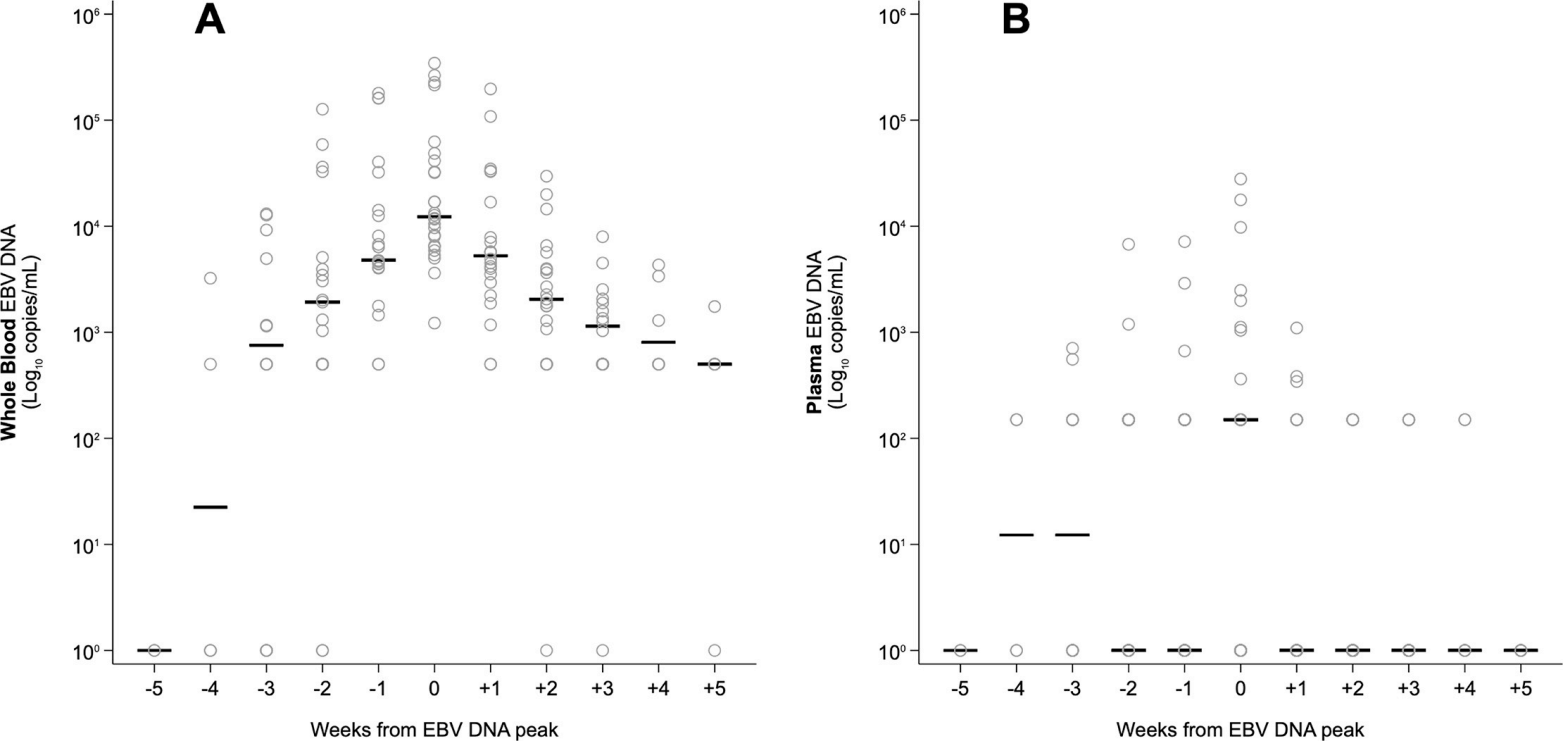
Distribution per week of EBV-DNA levels in WB and plasma samples with respect to EBV-DNA peak in WB, placed at T0. A: WB samples. B: plasma samples.

**Fig 8:**
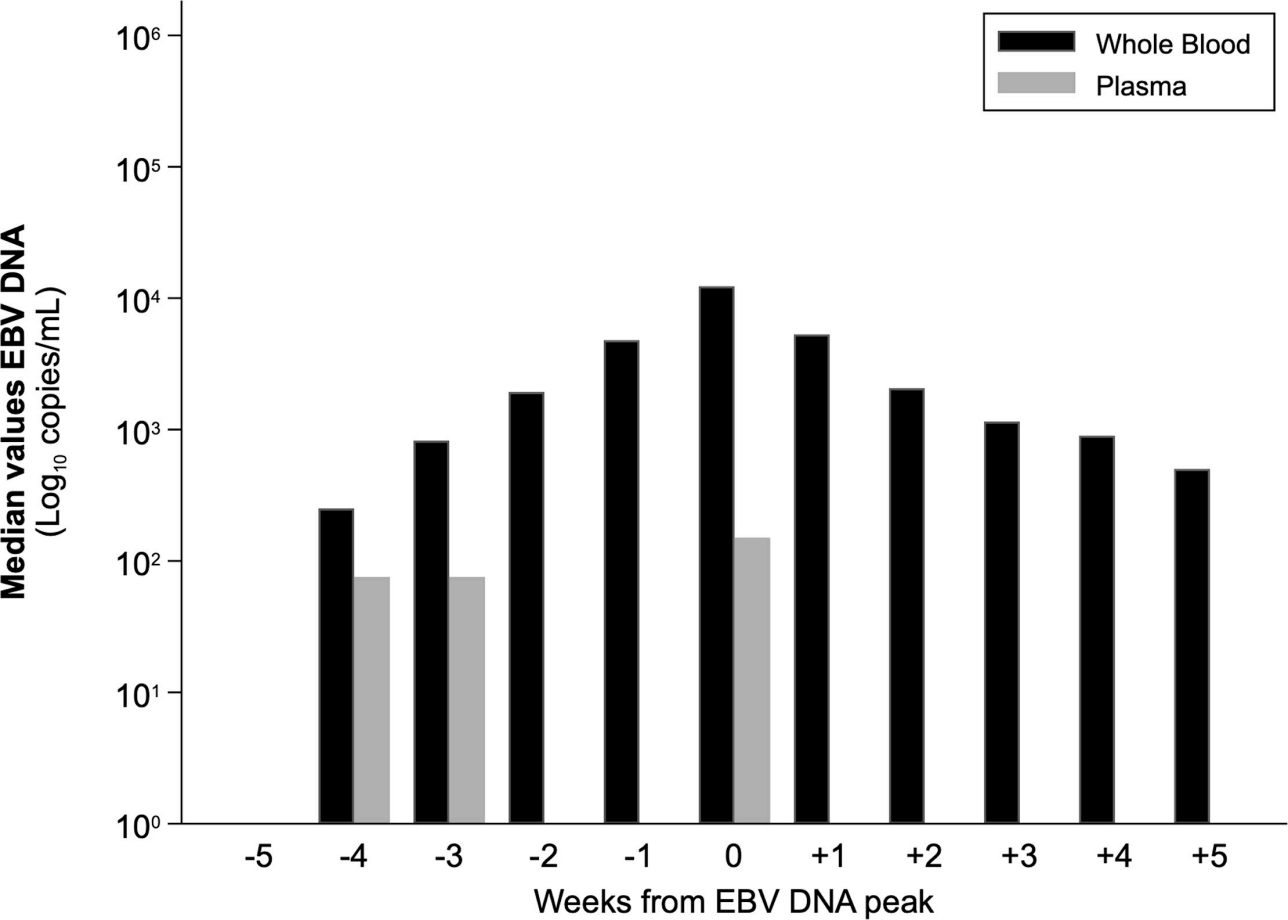
Median values of EBV-DNA peak in WB, placed at T0.

The distribution of EBV-DNA values with respect to WB peak time (T0) was dissimilar in the two blood compartments (Fig 7A and 7B). EBV-DNA peak levels were not reached simultaneously in WB and plasma in the majority of cases (18/26 active infection episodes; 69.2%). At T0, the median EBV-DNA load was equal to 12,285 copies/mL WB (range, 1,222–341,550) and below the LLoQ of the assay in plasma, i.e. 150 copies/mL (range, 150–27,900), resulting in a difference of 2.2 log_10_ copies/mL (p<0.001). In addition, at WB peak time, the 34.6% of the plasma samples (9/26 active infection episodes) resulted EBV-DNA negative. Finally, taking into account the median viral DNA levels, plasma samples tested EBV-DNA negative at most time-points. In the three time-points with a positive median plasma value, a positivity below the LLoQ was observed (Fig 8).

## Discussion

CMV and EBV-DNAemia detection and quantification by molecular assays during post-transplant period is the gold standard method to diagnose infection, guide preemptive strategies and monitor response to therapy [4,7]. The optimal blood compartment to test and the optimal viral load cut-off to use for initiating and interrupting preemptive therapy is unclear [4,7]. The present multicenter study analyzed, for the first time, the kinetics of both CMV and EBV-DNA in WB and plasma samples collected from adult KTRs during entire episodes of active infection by using a single CE-marked and FDA-approved automated molecular method. The correlation of virological results between the two blood sample types was also established. Notably, since a single approved method was used to test WB and plasma paired samples, the results obtained are not biased by inter-assay quantification variability due to the different analytical characteristics of the molecular assays (i.e. the limit of detection, the lower and upper limit of quantification, reproducibility and accuracy) that are known to affect the results [11]. The inter-operator reproducibility was also minimized since most samples were investigated in only one center. Finally, pre-analytical characteristics were consistent among the centers.

Regarding CMV infection, as previously observed in HSCT [6], despite a consistently high correlation between CMV-DNA levels in WB and plasma, differences in viral load values and in CMV-DNA kinetics in the two blood compartments were observed. Analyzing multiple complete infection episodes, although peak viral load was generally reached simultaneously in the two blood compartments, a higher percentage of CMV-DNA positive samples was observed in WB than in plasma in the ascending phase of infection. Although a more sensitive assay allows an earlier diagnosis of active CMV infection [12], no conclusions about the clinical advantage of the greater sensitivity for detection of CMV-DNA in WB than in plasma can be drawn from this study. With regard to viral load values, CMV-DNA in WB was constantly higher (about 1 log_10_) than in plasma since the onset of infection until the peak of viral load, after which it immediately decreased more rapidly than in plasma. At 3 weeks after the peak, viral load was similar in the two blood compartments; thus, only at this time the two measures could be considered equivalent. These findings confirm a more rapid initial decline in viral load in WB than in plasma, both in HSCT and SOT setting [6,12]. The CMV-DNA levels detected in plasma could represent free CMV-DNA released from cells and tissues [6,12]. In this regard, a recent study by Tong et al. observed that CMV-DNA in the plasma of SOT recipients is almost exclusively free DNA, highly fragmented and not virion associated [13]. As reported by the authors, their findings have implications for interpretation of dynamic changes in serial CMV loads [13]. In particular, in our cohort, in those patients who received antiviral therapy, the less steep trajectory of plasma viral load after the CMV-DNA peak could led to hypothesize a slower virological response to treatment than what could be inferred by analyzing the WB viral load trajectory. Moreover, if a plasma PCR assay had been used to guide the duration of the first course of valganciclovir/ganciclovir treatment, the length of antiviral therapy in about 3/4 of our patients would have been 7–14 days longer due to residual plasma CMV-DNAemia. Notably, since none of these patients developed a relapse of CMV infection during the follow-up period, the use of a WB PCR assay, avoiding unnecessary prolonged treatment, represents savings in terms of drug toxicity and cost of treatment. Unlike the early descending phase of CMV-DNAemia in which overlapping results were obtained [12], Lisboa et al. reported that CMV-DNA was detected more frequently in WB than in plasma at the end of antiviral therapy. Two different molecular assays were used by the authors and it can be speculated that among the reasons for the detection of residual WB CMV-DNAemia was a higher sensitivity of WB PCR assay than the plasma one [12,14,15]. No difference in the descending phase of infection between the two blood compartments was observed in the group of patients who did not require antiviral therapy. However, fewer untreated CMV infection episodes were available for this study.

The analysis of all WB and plasma sample pairs showed a lower correlation between EBV-DNA levels than CMV-DNA levels in the two blood compartments. EBV-DNAemia was more frequently quantified in WB than in plasma. Of note, EBV positive samples with a quantitative result in both blood compartments were observed only in 1/10 of cases. Furthermore, in 1/3 of the infection episodes, peak viral loads were detected in WB samples while their matched plasma samples were negative. Finally, by analyzing multiple complete infection episodes, in plasma, unlike in WB, it was not possible to observe an ascending phase, a peak and a descending phase of EBV-DNAemia. Plasma omits the presence of cell-associated virus which could explain the marked differences between WB and plasma viral loads and kinetics. Consistent with the literature in both HSCT and SOT [6,16–18], these findings showed the advantages of using WB PCR assay rather than plasma to diagnose active EBV infection and track viral replication in KTRs. Prior studies demonstrated that WB loads and peripheral blood mononuclear cells loads (considered an indirect measure of EBV-driven B-cell proliferation) correlate well and that WB is an acceptable alternative to testing peripheral blood mononuclear cells for quantifying EBV-DNA loads [16,17]. However, Kanakry and colleagues investigating the clinical significance of detecting EBV-DNA in plasma and in peripheral blood mononuclear cells of patients with or without EBV-related diseases, observed that plasma EBV-DNA was a good marker of EBV^+^ PTLD and performed better than cellular EBV-DNA as a marker of a broad range of EBV^+^ diseases [19].

In conclusion, our results suggest use of WB as specimen type for the post-transplant surveillance of both CMV and EBV infections and highlight that only one specimen type should be used in serial virological monitoring of each patient in order to ensure result comparability. In the near future, it is advised that clinical trials be carried out in large cohorts prospectively assessing whether, and to what extent, the different viral load kinetics in WB and plasma, as demonstrated in this preliminary study, could have implications on clinical intervention strategies; particularly for guiding anti-CMV therapy administration and identifying patients at higher risk of developing post-transplant EBV-related complications. It is also suggested that the kinetics of CMV and EBV-DNA in the two blood compartments according to the type of infection (first versus recurrent episodes of infection) be investigated.

The establishment of testing methods, frequency of testing and viral DNA thresholds for preemptive therapy management is an additional future challenge to be faced in order to obtain a standardized diagnostic and therapeutic approach in the transplant setting.

## Supporting information

S1 Table Induction and maintenance immunosuppressive therapy in the study cohort. (DOCX)

S1 Fig Ascending and descending linear slopes of viral load in WB and plasma, for patients who received basiliximab (BSX) or anti-thymocyte immunoglobulin (ATG) as induction therapy and a triple drug regimen consisting of tacrolimus, mycophenolate mofetil and steroids as maintenance therapy. (TIF)

S2 Fig ANOVA-estimated ascending and descending trajectories of viral load in WB and plasma, for patients who received basiliximab (BSX) or anti-thymocyte immunoglobulin (ATG) as induction therapy and a triple drug regimen consisting of tacrolimus, mycophenolate mofetil and steroids as maintenance therapy. (TIF)

S3 Fig Ascending and descending linear slopes of viral load in WB and plasma based on underlying disease. (TIF)
