# Supplementary material for: Kinetics of cytomegalovirus and Epstein-Barr virus DNA in whole blood and plasma of kidney transplant recipients: Implications on management strategies
Source: PLoS One. 2020 Aug 25;15(8):e0238062. doi: 10.1371/journal.pone.0238062 (PMC7447038; doi:10.1371/journal.pone.0238062)
Supplement: S1 Table — (DOCX) [file pone.0238062.s001.docx]

| **IMMUNOSUPPRESSIVE THERAPY**  **Induction^a^** – **Maintenance** | **IS Drugs plasma levels**  **range - ng/mL**  **< 2 month post-TX** | **IS Drugs plasma levels**  **range - ng/mL**  **3-6 months post-TX** | **IS Drugs plasma levels**  **range - ng/mL**  **7-12 months post-TX** |
| --- | --- | --- | --- |
| BSX – FK + steroids + MMF | 10-12 | 8-10 | 6-8 |
| ATG – FK + steroids + MMF | 10-12 | 8-10 | 6-8 |
| ATG **–** FK + steroids + EVR | FK 4-7; EVR 3-8 | FK 2-5; EVR 3-8 | FK 2-4; EVR 3-8 |
| BSX – FK + steroids + EVR | FK 4-7; EVR 3-8 | FK 4-7; EVR 3-8 | FK 4-7; EVR 3-8 |

IS: immunosuppressive; TX: transplant; BSX: basiliximab; ATG: anti-thymocyte immunoglobulin; FK: tacrolimus; MMF: mycophenolate mofetil; EVR: everolimus.

**^a^**Basiliximab (20 mg) during transplantation (day 0) and on day 4 or antithymocyte globulin (1.5 mg per kilogram of body weight daily) on day 0 and on days 1 through 4.
