# Supplementary figures and images for: Kinetics of cytomegalovirus and Epstein-Barr virus DNA in whole blood and plasma of kidney transplant recipients: Implications on management strategies
Source: PLoS One. 2020 Aug 25;15(8):e0238062. doi: 10.1371/journal.pone.0238062 (PMC7447038; doi:10.1371/journal.pone.0238062)

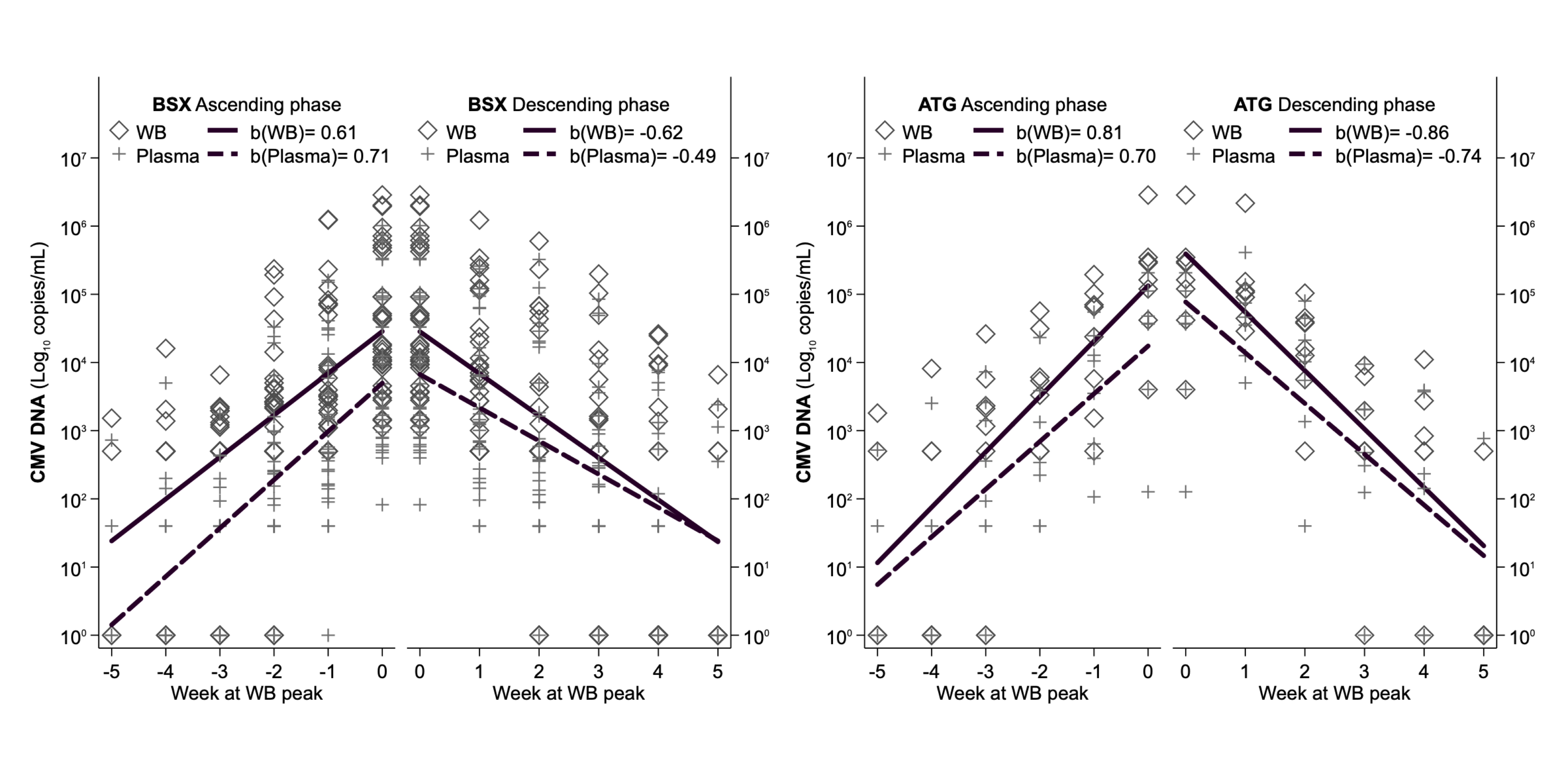

Supplement: S1 Fig — (TIF) [file pone.0238062.s002.tif]

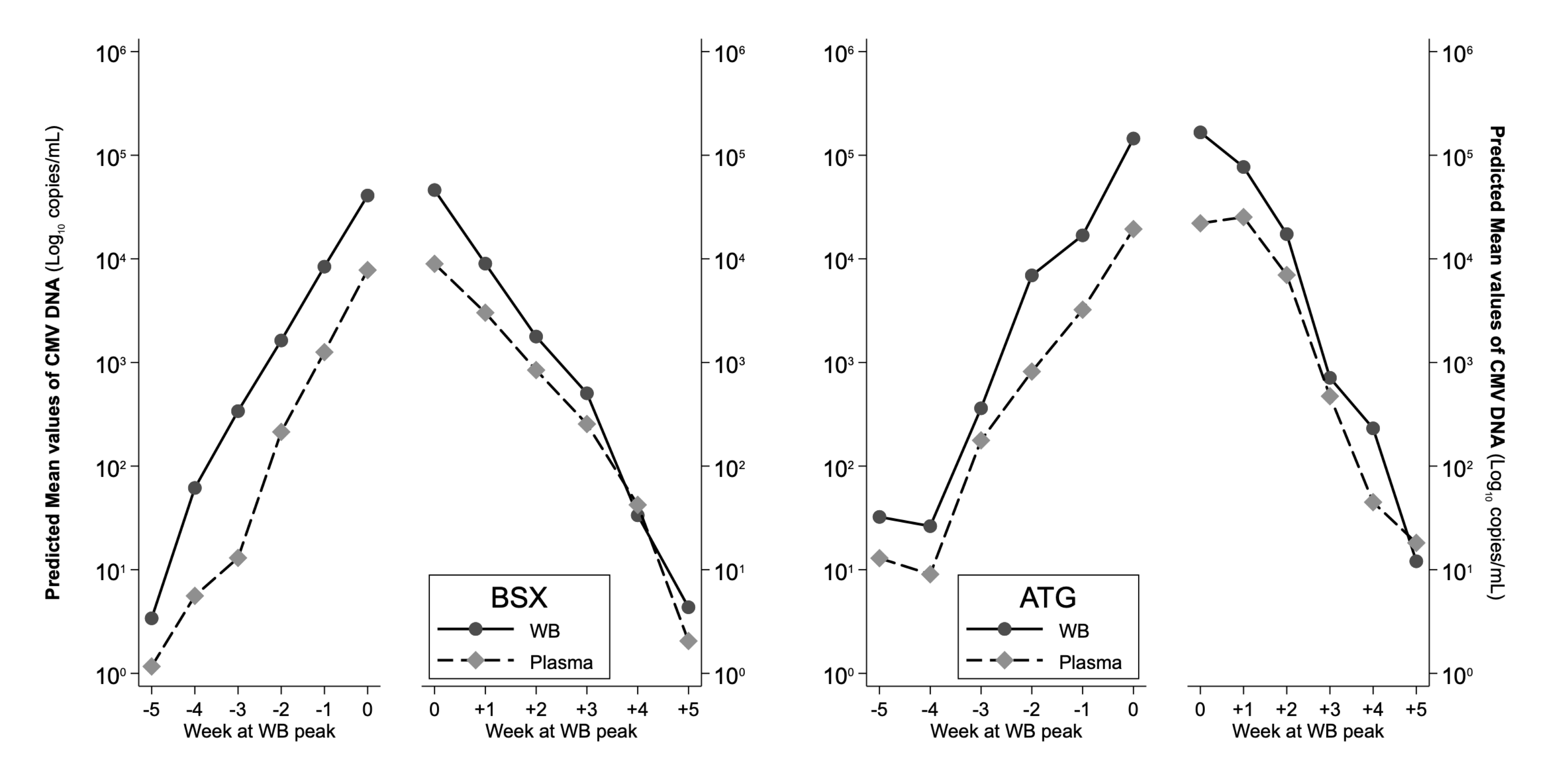

Supplement: S2 Fig — (TIF) [file pone.0238062.s003.tif]

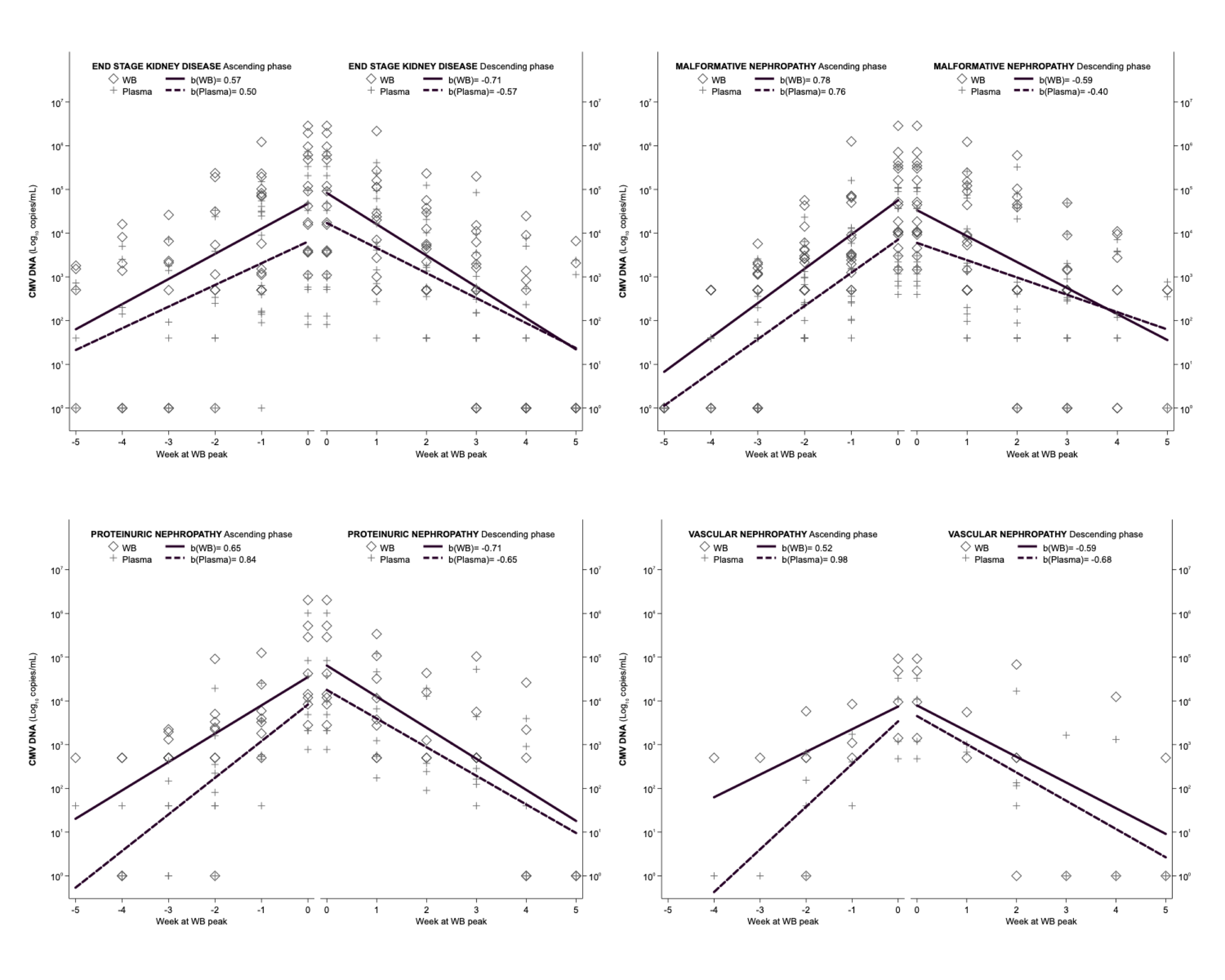

Supplement: S3 Fig — (TIF) [file pone.0238062.s004.tif]
